# Westminster Hospital

**Published:** 1830-10-01

**Authors:** 


### VIII.

### WESTMINSTER HOSPITAL.

#### I. CASE OF EXOSTOSIS TREATED WITH MERCURIAL FRICTION AND TURPENTINE.

ANN ROGERS, æt. 20, admitted July 15th, 1829, under Mr. Guthrie, with swellings on the shin-bone of the left leg—she is a tall and tolerably healthy looking woman, her face seems rather to express a scrophulous habit.

She states about four years ago she caught a very severe cold—she was menstruating at that period, and the cold affected her very violently—she had pains in her head and back, her whole body swelled and more particularly her legs, which pitted on pressure of the finger. She has never been quite well since that time—she is very positive in her assertion that she has never had any syphilitic affection. She is unmarried and lived as a servant. It nevertheless appears probable from collateral circumstances that there may have been some such cause for the swellings on her shin-bone. She suffers from continual pain down the tibia of the left leg, which increases in violence at night. It is about seven years since the catamenia commenced, and they have appeared at regular periods ever since. She attributes these swellings to having struck her leg against the stairs about the time when she first perceived them. She now complains of severe pain in the head and the shin of the left leg—pulse regular, 88 in the minute—tongue clean—bowels tolerably open.

℞ Hyd. Subm. gr. ij.
Pulv. Rhei, gr. xij. ft. pulv. bis in die sumend.

July 19th. For the last three nights three leeches have been applied on the swellings with some little benefit—Mr. Guthrie has ordered the following mixture.

℞ Ol. Terebinth. ℥ss.

Mucilag. q. s. ft. haust bis in die sumend.

23d. She complains of the medicine affecting her head—she is ordered the following plaster to be kept constantly applied over the tibia.

℞ Ung. Hyd. Fort.

Ext. Belladonnæ, āā. ℥i. ft. empl.

27th. She thinks she is worse since the application of the plaster—and that she felt more relief from the leeches than any thing else—she still complains of her head—Pil. Hyd. c. Col. gr. x. hac nocte—Haust. Aperiens cras mane.

August 12th. She has been rubbing in during the last ten days, and continues her medicine (turpentine) without much apparent benefit.

Aug. 29. She continues the mercurial friction—the lumps appear rather increased in size—complains of great pain in her head. C.c. nuchæ ad ℥x.

Sep. 10. No change—mouth not sore yet. Cap. pil. hyd. gr. v. o. n.

Oct. 4. The gums are very slightly affected. The leg is much better, but though the mercury has not made her mouth sore, its deleterious effects on the constitution are evident—she is pale, thin, and out of health. She is ordered to discontinue rubbing in—pills, &c.

15th. Since she has desisted rubbing in, she has complained of some slight return of pain in her shin-bone.

20th. Ordered a belladonna plaster to the left leg.

24th. Complains of great pain in her head—C. c, nuchæ ad ℥viij.

28th. Her leg is in less pain—the cupping relieved her head.

Nov. 2. Much the same.

℞ Ol. terebinth ℥j.

Tinct. lyttæ ♏. x.

Mucilag. gum acac. ℥j. ft. tinct. bis in die sumend.

9th. The medicine produces great irritation in the urinary organs, but her head is not much relieved—belladonna plaster has been renewed.

10th. Pain in the head very great—C. c. nuchæ ad ℥x.

16th. Irritation excited in the urinary organs has increased—she says she has passed blood—her head is much relieved.

24th. Severe pain in her head has returned from time to time, but upon the whole she is evidently much relieved. The tibia is much smoother and free from pain.

25th. She left the hospital to day at her own desire.

This is evidently one of those cases in which mercury, in whatever doses it may be employed, or however varied may be the mode of application, will not produce salivation. That it affected the constitution was, however, very apparent, and her health, after a time, declined rapidly, until its use was discontinued. She seemed at last, much benefited by the turpentine combined with the tinct. lyttæ.

### II. Diseased Testicle.—Extirpation—Cure.

James Thomson, æt. 32, admitted Dec. 23, 1829—with the integuments of the scrotum on the right side entirely destroyed, and a large irregular surface projecting out, which, on examination, proved to be the testicle. It is of a pale yellow colour, with here and there red granulations, and a few bands growing across. A red hollowed groove, leaving a white excavated edge, surrounds it. The surface of the sore is hard and not very painful on pressure.

Small pustules also have appeared within the last four days on the prepuce, and there now remain small white patches like flattened pustules.

He is a cabinet maker, and having lived in a wild irregular manner, he has suffered both from gonorrhœa and syphilis—repeatedly from the latter. About seven years ago he had a bubo. He traces the present disease back nine months, when he had a chancre on the gland—for which he made his mouth sore and applied the black wash—it healed in about a fortnight. Two weeks after this the testicle gave him great pain, and swelled to three or four times the natural size—he had six leeches and cold lotion applied, and since then, at intervals, several dozen of leeches—he did not however remain confined, but moved about with it supported by a suspensatory bandage—nor until a few days previous to his admission did he ever lay up.

About six weeks ago the skin appeared to be destroyed, and two or three small holes were observed in the scrotum, discharging matter—he had a poultice applied, and the holes gradually united and formed one large ulcer, from whence a very copious and offensive discharge proceeded—it has continued in much the same state up to the present time. He has generally enjoyed good health, with the exception of repeated attacks of syphilis. And during the whole of the last nine months, in which the disease has been advancing, he has felt very well, and suffered but little pain from the diseased testicle, except lately, that he has experienced a twitching, prickly sensation in the lower extremity of that side. His appetite has been good, and he has followed his business, till within the last ten days—he is pale, but not very unhealthy in appearance. His pulse is full, regular, and natural—bowels open; tongue clean.

Dec. 20. He continues his poultice, and suffers little pain.

Jan. 3. A powder, composed of equal parts of myrrh and lapis calaminaris, is ordered to be applied to the ulcerated surface daily.

10th. The appearance of the testicle is rather improved.

Feb. 10th. The powder has been applied daily, and the sore is much diminished in size—nearly one-half; it remains, however, much the same in character. General health unimpaired.

He continued in the same state until the 10th of April, when Mr. Harding proceeded to the operation for castration. A straight incision was made, which separated the chord from its sheath and divided it. In consequence of the adhesions produced by the disease, the testicle could not be separated from the scrotum, without the aid of the knife. Two arteries were taken up—and the hæmorrhage during the operation was very trifling—the edges were brought together with three sutures. About an hour after he had been in bed—hæmorrhage came on, which could not be stopped without opening the wound, a large coagulum was then taken out of some ounces weight, and after a careful search, the cause of the hæmorrhage was found to be the arteria media scroti, very much enlarged. This was secured, and the bleeding entirely ceased. About a week after this there was considerable tendency to inflammation, which was relieved by leeches and cold lotion. The healing process then went on favorably—and the patient left the hospital quite well.

III. CASE OF TRISMUS TRAUMATICUS, ARISING FROM DECAYED TEETH AND COLD.

Caroline Lewis, æt. 24, admitted Dec. 21, 1829, under Mr. Lynn, with her jaws firmly locked, complaining of great pain in her head and neck. It appears that the jaws closed suddenly one afternoon, (three days before her admission) and resisted all her efforts to open them. She had suffered from a cold for a fortnight before, which had swelled her face inside and out, accompanied with most violent tooth-ache. From her earliest recollection she has been troubled with frequent and severe attacks of tooth-ache, scarcely ever a month free. She menstruates regularly, but scantily. Two or three of her side teeth are, fortunately, reduced to mere stumps, so that she can swallow any liquid through the cavities they have left.

Dec. 21st.

℞. Hydr. Submur. gr. iv.

Pulv. Jalapæ . ʒss. Ft. pulv. st. sumend.

℞. Liq. Ant Tart .... ʒj.

Liq. Ammon. Acet. ℥iij.

Mist. Camph. .... ℥v. Ft. mist et cap. coch. iij. 4tis horis. V.S. ad ℥xij. and fomentation to the cheeks.

22d. There is less fever, and also some slight abatement of pain in the head and face. Continue fomentation to the face and medicine.

23d. Much the same. Bowels freely opened.

℞. Lin. Volatil. c. ℥ij. pro fotu. Two eggs per diem.

24th. She was much the same all the morning, but complained of sickness. Towards evening it increased, and a violent fit of vomiting came on; she felt choking, when she felt a "crunching," attended with violent pain, and her mouth opened.

25th. She feels much better—pain in the face less.

28th. The glands on the right side of the neck are rather enlarged, and her face is not quite free from pain. On examining her mouth, the whole of her teeth, with the exception of the three or four front ones, are in a state of decay. Most of those that remain being merely blackened stumps; tongue tolerably clean; bowels confined.

Jan. 3d. Dismissed well. She refused to have any thing done to her teeth.

### CLINICAL REVIEW.

### XLIV.

### WESTMINSTER HOSPITAL.

Ulceration of the Tonsils—Death. Sectio Cadaveris.

William Pimlett, æt. 51, admitted April 24th, 1830, under Dr. Roe. Complains of severe cough and great difficulty of swallowing in consequence of a sore throat. He is very much emaciated, his pulse slow and feeble, countenance anxious, and the surface of his body cold. He is a coal-porter, and states that four months ago he caught a severe cold, attended with cough and dyspnœa, and two months afterwards he felt his throat affected. Opening medicines were administered, and he used gargles, but he felt himself grow worse. Three weeks ago the disease had advanced so far that it was with the greatest difficulty he could swallow any thing, and he became in consequence much debilitated. His cough has become more frequent lately, and expectoration plentiful. He does not sleep at night, and his appetite is impaired. Immediately on his admission he was ordered the warm bath.

29th. Passed a tolerable night; there is less anxiety of countenance—pulse full and quick—tongue clean—bowels confined. Haust. aperiens st.

℞. Pulv. hyd. cum cretà,
Ipecac. c. āā gr. v.
ft. pulv. bis in die sumendus.

℞. Decoct. cinchon. ℥j.

Acid. nitr. ♏. x.—ter in die.

C. c. jugulo ad ℥vj.—Arrow-root and milk daily.

30th. Slept well during the night—has less pain in the throat, and not so much difficulty in swallowing—cough less frequent—pulse softer—bowels open.

Moxa applicet. jugulo.

May 1st. Hirudines xij. jugulo et rep. moxa.

May 4th. He slept better last night, and has less difficulty in swallowing—there is also less anxiety of countenance, but he complains much of debility.—Pulse is feeble but regular. Tongue clean—bowels confined. Haust. purg. st. et cont. mist.

May 6th. The moxa was re-applied to-day. He is ordered beef-tea.

8th. He complains that he experiences great pain in swallowing the medicine. The dose of nitric acid is diminished to ♏. v.

℞. Pil. hyd.
Pulv. opii, āā gr. j.—bis in die.

10th. He is evidently worse—the difficulty of swallowing is increased, and his countenance expresses great anxiety. Pulse very feeble, surface of the body cold. A tube was introduced into the œsophagus through the nostrils and four ounces of wine administered; from the great irritation produced by the tube it was obliged to be withdrawn before more could be passed. Two pints of milk boiled up with two ounces of arrow-root, and the same quantity of port wine were thrown up the rectum this morning, and repeated again in the evening.

11th. He appears to have rallied again. He enjoyed a little sleep in the night, and his pulse has gained a little power. Tongue clean—bowels open—countenance less pale and anxious. He has attempted to swallow some beef-tea, and a tube has been passed into the œsophagus, and about half a pint of strong beef-tea introduced.

12th. He swallowed nearly a pint of the arrow-root last night by the natural efforts. He articulates more distinctly, but his pulse is still feeble. He has inhaled the fumes of the factitious linaria twice, and is to repeat it daily.

13th. He is evidently sinking, and is unable to swallow any thing.

14th. He died this afternoon.

16th. *Sectio Cadaveris.* The body presented a very emaciated appearance. On examining the thorax the lungs were found adherent throughout the surface to the sides. The upper and middle lobes were completely tuberculated, and the inferior approaching to a state of hepatization. In the superior parts the tubercles were in a state of suppuration, and in the left apex there was a small vomica.

The mucous membrane of the bronchiæ and trachea was natural—the bronchial tubes were nearly filled with a muco-purulent fluid—the whole interior surface of the larynx presented a granulated surface, which had evidently secreted a quantity of pus. The ligamentous portions of the larynx were much thickened, and there appeared a general amalgamation of the tissues. The epiglottis especially was much thickened, and the posterior portion enlarged and uneven.

Abdomen healthy.

